# Genetic associations between gut microbiota and type 2 diabetes mediated by plasma metabolites: a Mendelian randomization study

**DOI:** 10.3389/fendo.2024.1430675

**Published:** 2024-08-09

**Authors:** XuWen Zheng, MaoBing Chen, Yi Zhuang, Liang Zhao, YongJun Qian, Jin Xu, JinNuo Fan

**Affiliations:** Emergency Department, Wujin People’s Hospital Affiliated with Jiangsu University and Wujin Clinical College of Xuzhou Medical University, Changzhou, Jiangsu, China

**Keywords:** type 2 diabetes, gut microbiota, plasma metabolites, Mendelian randomization, linkage disequilibrium score regression, meta-analysis

## Abstract

**Background:**

Numerous research studies have indicated a possible association between type 2 diabetes (T2DM) and gut microbiota. To explore specific metabolic pathways connecting gut microbiota and T2DM, we employed Mendelian randomization (MR) and linkage disequilibrium score regression (LDSC) techniques.

**Methods:**

This research utilized data from genome-wide association studies (GWAS) that are publicly accessible. We evaluated the genetic correlation between gut microbiota and T2DM using LDSC. Causality was primarily determined through the inverse variance weighted (IVW) method. To verify the robustness of our results, we conducted sensitivity analyses using several approaches, including the weighted median, MR-Egger, and MR-PRESSO. We integrated summary effect estimates from LDSC, along with forward and reverse MR, into a meta-analysis for T2DM using various data sources. Additionally, mediation analysis was performed to explore the impact of plasma metabolites on the relationship between gut microbiota and T2DM.

**Results:**

Our study indicated a significant genetic correlation between genus *RuminococcaceaeUCG005* (Rg = −0.26, Rg_P = 2.07×10^−4^) and T2DM. Moreover, the forward MR analysis identified genus *RuminococcaceaeUCG010* (OR = 0.857, 95% CI 0.795, 0.924; P = 6.33×10^−5^) and order *Clostridiales* (OR = 0.936, 95% CI 0.878, 0.997; P = 0.039) as being significantly associated with a decreased risk of T2DM. The analysis also highlighted several plasma metabolites as significant mediators in these relationships, with metabolites like octadecadienedioate (C18:2-DC) and branched chain 14:0 dicarboxylic acid being notably involved.

**Conclusion:**

The findings demonstrate a significant impact of gut microbiota on T2DM via plasma metabolites, suggesting potential metabolic pathways for therapeutic targeting. This study enhances our understanding of the microbiota’s role in T2DM pathogenesis and supports the development of microbiota-based interventions.

## 1 Introduction

Type 2 diabetes (T2DM) affects over 400 million individuals globally, with prevalence rates continuing to soar alongside rising obesity levels and sedentary lifestyles. The disease disproportionately impacts aging populations, but recent trends show increasing incidence in younger demographics as well, attributed to lifestyle changes and genetic predispositions (1). The clinical presentation of T2DM can vary but commonly includes symptoms like polyuria, polydipsia, and unexplained weight loss. Additionally, T2DM leads to significant healthcare costs and productivity losses due to complications such as neuropathy, nephropathy, and retinopathy (2–4). Its pathogenesis involves a combination of genetic predisposition and environmental factors, leading to insulin resistance and β-cell dysfunction (3, 5, 6).

Among the most promising domains in T2DM research is the involvement of the microbiome as a potential environmental contributor. Recent research has highlighted the complex relationship between gut microbiota and T2DM. Observational studies have found significant correlations between gut microbiota diversity and T2DM (7, 8), while mendelian randomization (MR) studies have identified specific microbial genera such as *Bifidobacterium* and *Lachnoclostridium*, that may causally impact T2DM risk (9, 10).

Despite these advances, a significant research gap remains in understanding the precise mechanisms through which gut microbiota and plasma metabolites influence T2DM. To address this gap, we employed a combination of Linkage Disequilibrium Score Regression (LDSC) and mediation MR. The use of genetic variations as instrumental variables (IVs) is central to the practice of MR, an epidemiological method aimed at improving the reliability of causal conclusions (11). This approach provides two main advantages: it helps to overcome the issue of confounding variables and reduces the possibility of reverse causation, mainly because genetic variants are allocated randomly at the time of conception (11). LDSC is notable for its ability to evaluate genetic correlations using summary statistics from GWAS without being affected by overlapping samples (12). The primary aim of this research is to delineate the pathways by which gut microbiota influence T2DM through specific plasma metabolites using an MR framework, thereby providing a clearer picture of the disease’s etiology and pointing towards targeted interventions.

Understanding how gut microbiota and specific metabolites influence T2DM can lead to new therapeutic strategies and improve prevention and treatment efforts, ultimately reducing the global burden of the disease. By focusing on the mediating role of plasma metabolites, this study not only seeks to bridge the gap between genetic predispositions and microbial influences but also aims to uncover specific metabolic pathways that could be targeted for therapeutic intervention.

## 2 Methods

### 2.1 Study design

**Figure 1:**
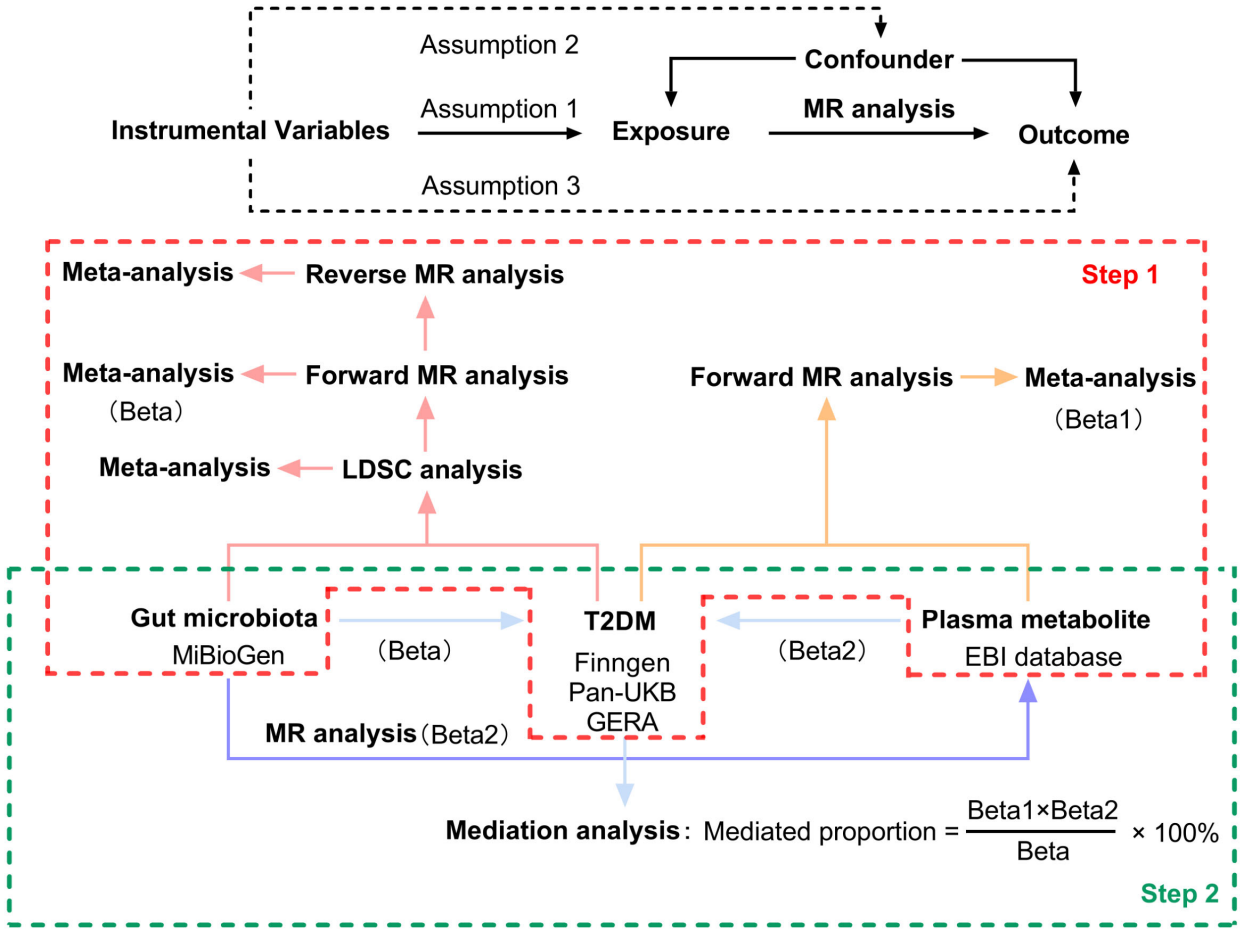
Three assumptions of MR analysis and overview of the study design. MR, mendelian randomization; LDSC, Linkage Disequilibrium Score Regression; T2DM, type 2 diabetes.

This study made use of publicly available GWAS data, applying IVs that met three essential criteria necessary for conducting MR analysis (1): The genetic variants used as instruments must demonstrate a significant association with the exposure under investigation; (2) These variants must not have any associations with other potential risk factors for the outcome; and (3) The effect of the genetic variants on the outcome must occur solely through the exposure (11). **Figure 1** illustrates the overarching design of this investigation. In the first step, we conducted LDSC, forward, and reverse MR analyses examining the relationship between 211 gut microbiomes and T2DM. Additionally, we performed forward MR analyses involving 1,400 plasma metabolites associated with the disease. Outcome data for T2DM were sourced from three distinct databases. Meta-analysis integrated the summary effect estimates from LDSC, forward MR, and reverse MR to assess T2DM across various data sources. In the second step, MR analyses were performed between the identified gut microbiomes and the identified plasma metabolites. The indirect effects (IE) of the identified gut microbiomes on T2DM via plasma metabolites was assessed using the product of coefficients method (13). All studies included in the analysis were approved by their respective institutional review boards and ethical committees, and consent forms were obtained from all participants.

### 2.2 Instrumental variable selection

The MiBioGen consortium conducted the largest genome-wide meta-analysis to date, identifying genetic variations that influence gut microbiota composition (14). This analysis involved 18,340 participants across 24 cohorts, with the majority being of European origin (n = 13,266). The MiBioGen database revealed 211 gut microbiota taxa, which included 12 unknown genera and 3 unknown families. Despite their minor representation, these unknown taxa were not excluded from our analysis, though results pertaining to these unidentified bacterial taxa will not be reported. Additionally, GWASs were performed on 1,091 metabolites and 309 metabolite ratios involving 8,299 participants from the Canadian Longitudinal Study on Aging (CLSA) cohort (15). To align with the first assumption of MR analysis, a significance threshold of P < 1×10^−5^ was employed for gut microbiota and plasma metabolites identified through GWASs, acknowledging that this rarely meets the genome-wide significance threshold (P < 10^−8^) (16). Furthermore, to adhere to MR’s requirement of no linkage disequilibrium (LD) among IVs, IVs were selected based on R^2^ < 0.001 and a clumping distance of 10,000 kb to maintain independent single nucleotide polymorphisms (SNPs). To mitigate the influence of weak IVs, the F-statistic (F = beta²/se²) was calculated for each SNP, discarding IVs with an F-statistic < 10 as weak (17, 18). Harmonization of SNPs in both the exposure and outcome datasets was performed to match alternative and reference alleles, thus eliminating SNPs with mismatched alleles to reduce inconsistencies. Ambiguous palindromic SNPs with minor allele frequencies close to 0.5 were excluded from the MR analyses. For the second assumption, MR-Egger intercept test and MR pleiotropy residual sum and outlier (MR-PRESSO) test were conducted to identify pleiotropy, excluding MR estimates with significant pleiotropy from the meta-analysis (P for intercept < 0.05 or P for global test < 0.05). Lastly, for the third assumption, SNPs significantly associated with the outcome (P < 1×10^−5^) were omitted from the MR analysis to ensure the validity of causal inferences. The IVs associated with all gut microbiota taxa and plasma metabolites were comprehensively listed in **Supplementary Tables 1**, **2**.

### 2.3 T2DM data sources

Summary-level data for T2DM were derived from three major sources: the Pan-UKB GWAS Version 0.4, released on March 16, 2023 (19); the FinnGen GWAS Release 10, released on December 18, 2023 (20); and the Genetic Epidemiology Research on Aging (GERA) (21). The total sample size encompassed 72,194 cases and 784,605 controls of European ancestry. The Pan-UKB GWAS utilized data from the UK Biobank, an extensive open-access database containing genotype information for hundreds of thousands of individuals, alongside with electronic health records and survey responses, aimed at studying populations of diverse ancestries (19). The FinnGen GWAS represents a comprehensive national genetic study, integrating genetic data with electronic health records (20). The GERA cohort, focused on age-related diseases with an average participant age of 63, is well-equipped to study a wide variety of clinically defined age-related conditions (21). Detailed descriptions of sample sizes, adjustments, and diagnostic criteria used in these studies are provided in **Table 1**.

**Table 1:** Detailed information on used summary-level data.

Exposure or outcome	Database	Participants included in analysis	Adjustments	ICD	Web source
Gut microbiota	MiBioGen	18,340 multiple-descent individuals	age, sex, technical covariates and genetic principal components		https://mibiogen.gcc.rug.nl/
Plasma metabolite	EBI database	8,299 European individuals	age, sex, hour since last meal or drink, genotyping batch and the first ten genetic principal components		https://www.ebi.ac.uk/gwas/
T2DM	FinnGen	42,593 cases and 337,038 controls of European ancestry	sex, age, genotyping batch and ten principal components	ICD-10: E11; ICD-9: 250.A	https://r10.finngen.fi/
	Pan-UKB	22,634 cases and 397,897 controls of European ancestry	sex, age, genotyping array, and the first 8 principal components	ICD-10: E11	https://pan.ukbb.broadinstitute.org/downloads/
	GERA	6,967 cases and 49,670 controls of European ancestry	seven derived principal components, sex, and age	ICD-9: 250.00, 250.02, 250.10, 250.12, 250.20, 250.22, 250.30, 250.32, 250.40, 250.42, 250.50, 250.52, 250.60, 250.62, 250.70, 250.72, 250.80, 250.82, 250.90, 250.92	http://cg.bsc.es/gera_summary_stats/

### 2.4 Statistical analysis

We conducted an analysis to explore the genetic correlation between gut microbiota and T2DM utilizing LDSC. To refine the GWAS summary data, we used HapMap3 references, excluding non-SNP variants like insertions and deletions (indels), as well as SNPs with ambiguous strand orientation, duplicates, or a minor allele frequency below 0.01. LDSC is adept at determining genetic correlations using GWAS summary statistics. It assesses the relationship between LD and test statistics to identify whether observed inflation is due to genuine polygenic signals or other biases (12). This approach is unaffected by sample overlap (22). Genetic covariance is calculated by multiplying the z-scores of variants associated with Trait 1 by those associated with Trait 2, and subsequently regressing these products against the LD score (23). After adjusting this covariance by SNP heritability, the genetic correlation becomes clear. Estimates of genetic correlation between gut microbiota and T2DM from three data sources were combined through fixed-effects meta-analysis.

MR analyses were performed to examine the relationships between gut microbiota and T2DM, plasma metabolites and T2DM, as well as gut microbiota and plasma metabolites. The primary MR estimate was calculated using the inverse variance weighted (IVW) method within a random-effects framework for causal analysis. The IVW method is best used when the MR assumptions are believed to hold true across all genetic variants. It provides the most precise estimate when there is no horizontal pleiotropy (11). To detect horizontal pleiotropy and ensure the reliability of our data, we utilized three sensitivity analyses: the weighted median, MR-Egger, and MR-PRESSO. The weighted median method is particularly useful when there is concern that some genetic variants may be invalid instruments due to pleiotropy. It provides a robust estimate that is less sensitive to invalid instruments compared to the IVW method (24). MR-Egger is particularly useful when there is concern about directional pleiotropy. It provides a more conservative estimate and tests for the presence of pleiotropy through the intercept term. If the intercept is significantly different from zero, this indicates the presence of directional pleiotropy (25). MR-PRESSO is best used when there is evidence or suspicion of pleiotropy. It improves the reliability of causal estimates by removing the influence of outlier variants that violate the exclusion restriction assumption (26). SNP heterogeneity was evaluated using the Cochran Q value. The MR-Egger intercept test was employed to detect horizontal pleiotropic effects. Combined estimates from IVW and sensitivity analyses were integrated using fixed-effects meta-analysis. Exposures represented by fewer than four SNPs were omitted from the analysis, as MR-PRESSO requires a minimum of four instrumental SNPs. Estimates indicating significant pleiotropy (P for intercept test < 0.05 or P for global test < 0.05) were also excluded from the meta-analysis.

A two-step MR analysis assessed if plasma metabolites mediated the influence of identified taxa on T2DM (27). To streamline the process, initial MR analyses were conducted between plasma metabolites and T2DM, followed by analyses between identified gut microbiomes and plasma metabolites. The IE of the gut microbiome on T2DM via plasma metabolites was assessed using the product of coefficients method (13). To calculate the mediated proportion of T2DM effect by plasma metabolites, the IE was divided by the total effect (28).

Bonferroni’s correction was applied separately to both LDSC and MR analyses in the meta-analyses to minimize the false discovery rate (29). LDSC correlations with p-values between 3.65×10^−4^ (0.05/137) and 0.05 were suggestive, while those with p-values less than 3.65×10^−4^ were significant. MR associations between gut microbiota and T2DM were suggestive if IVW p-values were between 2.37×10^−4^ (0.05/211) and 0.05, and significant if p-values were less than 2.37×10^−4^ or if both IVW and LDSC p-values were less than 0.05. MR associations between plasma metabolites and T2DM were suggestive if IVW p-values ranged from 3.57×10^−5^ (0.05/1400) to 0.05, and were considered significant if p-values were less than 3.57×10^−5^. All statistical analyses were performed using R software (version 4.3.1), utilizing the TwoSampleMR, GenomicSEM, and meta packages.

## 3 Results

### 3.1 LDSC analysis between gut microbiota and T2DM

**Figure 2:**
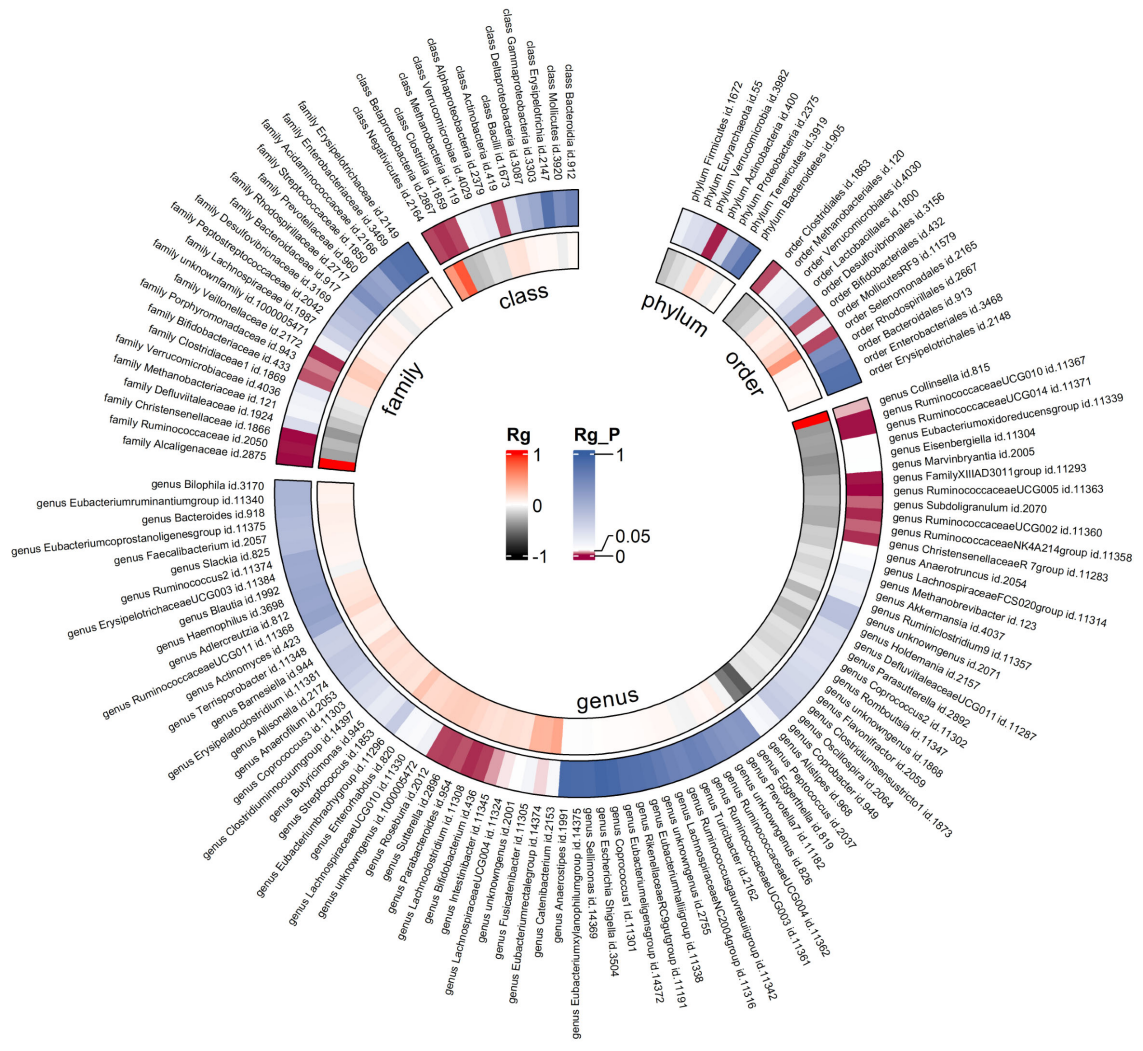
Circular heat map of meta-analysis of genetic correlation between gut microbiota and T2DM. Rg, estimate of genetic correlation; Rg_P, p-value for Rg.

Due to constraints like low heritability and small sample sizes, certain bacterial taxa are not suitable for the analysis mentioned above. We performed a meta-analysis of LDSC to evaluate the genetic correlation between 137 gut microbes and T2DM, including 7 unknown taxa (**Figure 2**). As shown in **Table 2**, LDSC showed a significant negative correlation between genetically predicted T2DM and genus *RuminococcaceaeUCG005* (Rg = −0.26, 95% CI −0.39, −0.12; Rg_P = 2.07×10^−4^). Furthermore, we identified a suggestive genetic correlation between genetically predicted T2DM and genus *RuminococcaceaeUCG010* (Rg = −0.37, 95% CI −0.59, −0.14; Rg_P = 1.19×10^−3^), order *Clostridiales* (Rg = − 0.23, 95% CI −0.41, −0.06; Rg_P = 0.010), genus *Parabacteroides* (Rg = 0.21, 95% CI 0.05, 0.38; Rg_P = 0.012), family *Porphyromonadaceae* (Rg = 0.15, 95% CI 0.02, 0.27; Rg_P = 0.022), genus *Sutterella* (Rg = 0.25, 95% CI 0.07, 0.44; Rg_P = 0.008), genus *Lachnoclostridium* (Rg = 0.17, 95% CI 0.05, 0.28; Rg_P = 0.005) and other 25 taxa. No heterogeneity or mild heterogeneity was observed across most of the results. Detailed information regarding all genetic correlation results is listed in **Supplementary Table 3**.

**Table 2:** Meta-analysis of genetic correlation between gut microbiota and T2DM from three large databases.

Exposure	Rg	Rg_Se	Rg_P	I^2^	P_heterogeneity
genus RuminococcaceaeUCG005	-0.258	0.070	0.0002	0	0.570
family Christensenellaceae	-0.248	0.071	0.0005	0	0.818
family Alcaligenaceae id.2875	1.018	0.296	0.0006	0	0.988
genus RuminococcaceaeUCG010	-0.366	0.113	0.0012	0.046	0.350
genus RuminococcaceaeUCG014	-0.331	0.103	0.0013	0	0.782
family Ruminococcaceae id.2050	-0.330	0.104	0.0015	0.287	0.246
genus FamilyXIIIAD3011group	-0.271	0.086	0.0017	0.656	0.055
phylum Actinobacteria	0.223	0.074	0.0028	0	0.681
genus RuminococcaceaeUCG002	-0.289	0.100	0.0040	0.571	0.097
genus Lachnoclostridium	0.168	0.059	0.0047	0	0.565
family Veillonellaceae	0.267	0.095	0.0051	0	0.865
genus ChristensenellaceaeR 7group	-0.173	0.062	0.0051	0	0.650
class Betaproteobacteria	0.814	0.293	0.0055	0	0.872
genus Sutterella	0.251	0.094	0.0077	0	0.763
genus Bifidobacterium	0.152	0.057	0.0082	0	0.892
genus Roseburia	0.260	0.099	0.0087	0	0.586
class Negativicutes	0.528	0.203	0.0094	0	0.491
order Selenomonadales	0.528	0.203	0.0094	0	0.491
class Clostridia	-0.237	0.092	0.0097	0.225	0.275
class Actinobacteria	0.136	0.053	0.0102	0	0.690
order Clostridiales	-0.232	0.090	0.0102	0.202	0.286
family Bifidobacteriaceae	0.140	0.056	0.0123	0	0.775
order Bifidobacteriales	0.140	0.056	0.0123	0	0.775
genus Parabacteroides	0.212	0.085	0.0124	0	0.891
genus Subdoligranulum	-0.238	0.098	0.0156	0.102	0.328
genus RuminococcaceaeNK4A214group	-0.292	0.121	0.0159	0	0.983
family Porphyromonadaceae	0.148	0.065	0.0222	0	0.803
genus Intestinibacter	0.174	0.080	0.0286	0	0.765
genus Collinsella	1.165	0.550	0.0340	0	0.715
genus Eubacteriumrectalegroup	0.377	0.185	0.0410	0	0.967
genus LachnospiraceaeUCG004	0.165	0.082	0.0443	0	0.704
genus Anaerotruncus	-0.183	0.093	0.0495	0.182	0.295

### 3.2 Forward MR analysis between gut microbiota and T2DM

**Figure 3:**
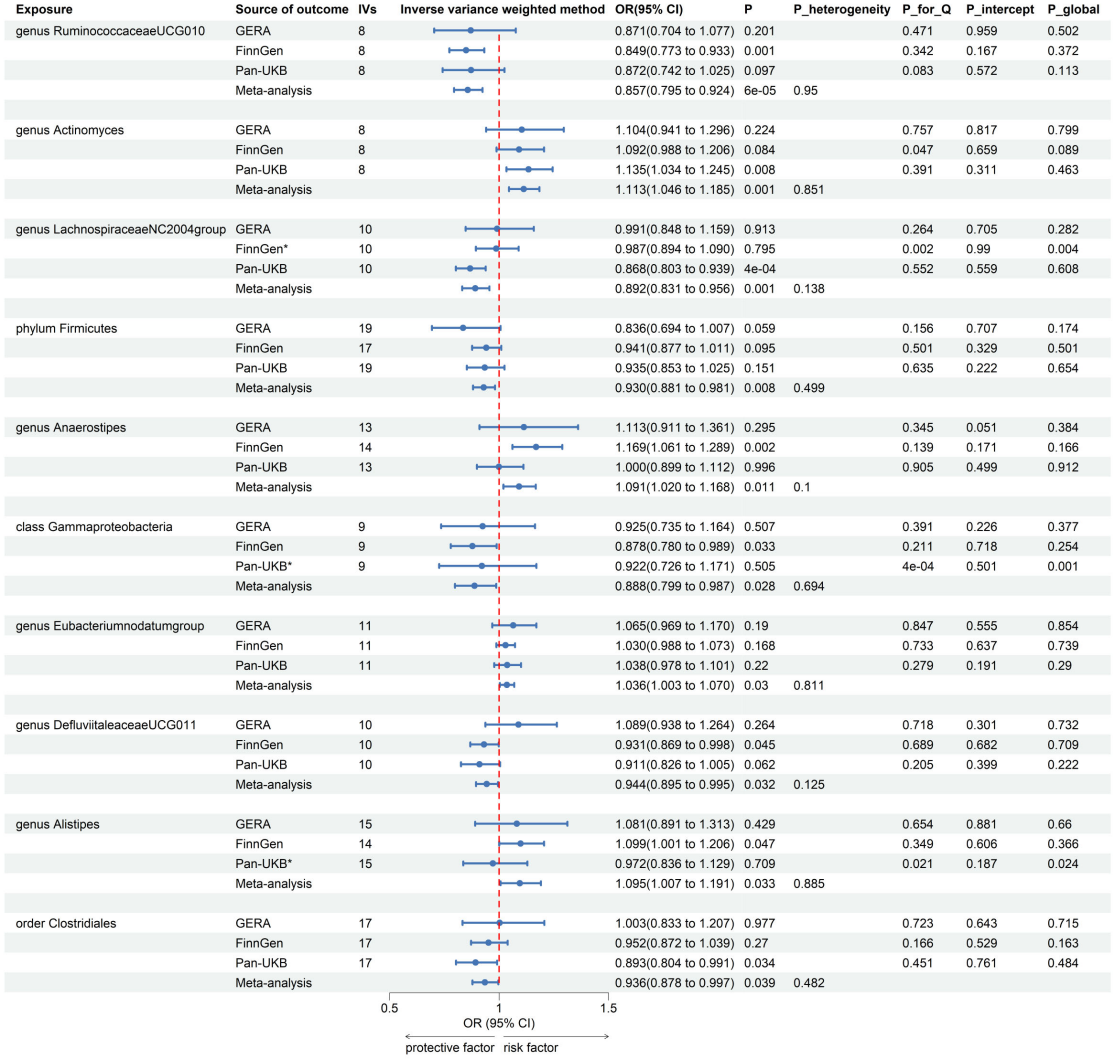
Forest plot of forward MR analysis between gut microbiota and T2DM. IVs, instrumental variables; CI, confident interval; P_heterogeneity, p-value of heterogeneity for meta-analysis; P_for_Q, p-value for Cochran Q test; P_intercept, p-value for MR-Egger intercept test; P_global, p-value for Global test; *, excluded from the meta-analysis due to SNPs less than 4 or significant pleiotropy.

**Figure 4:**
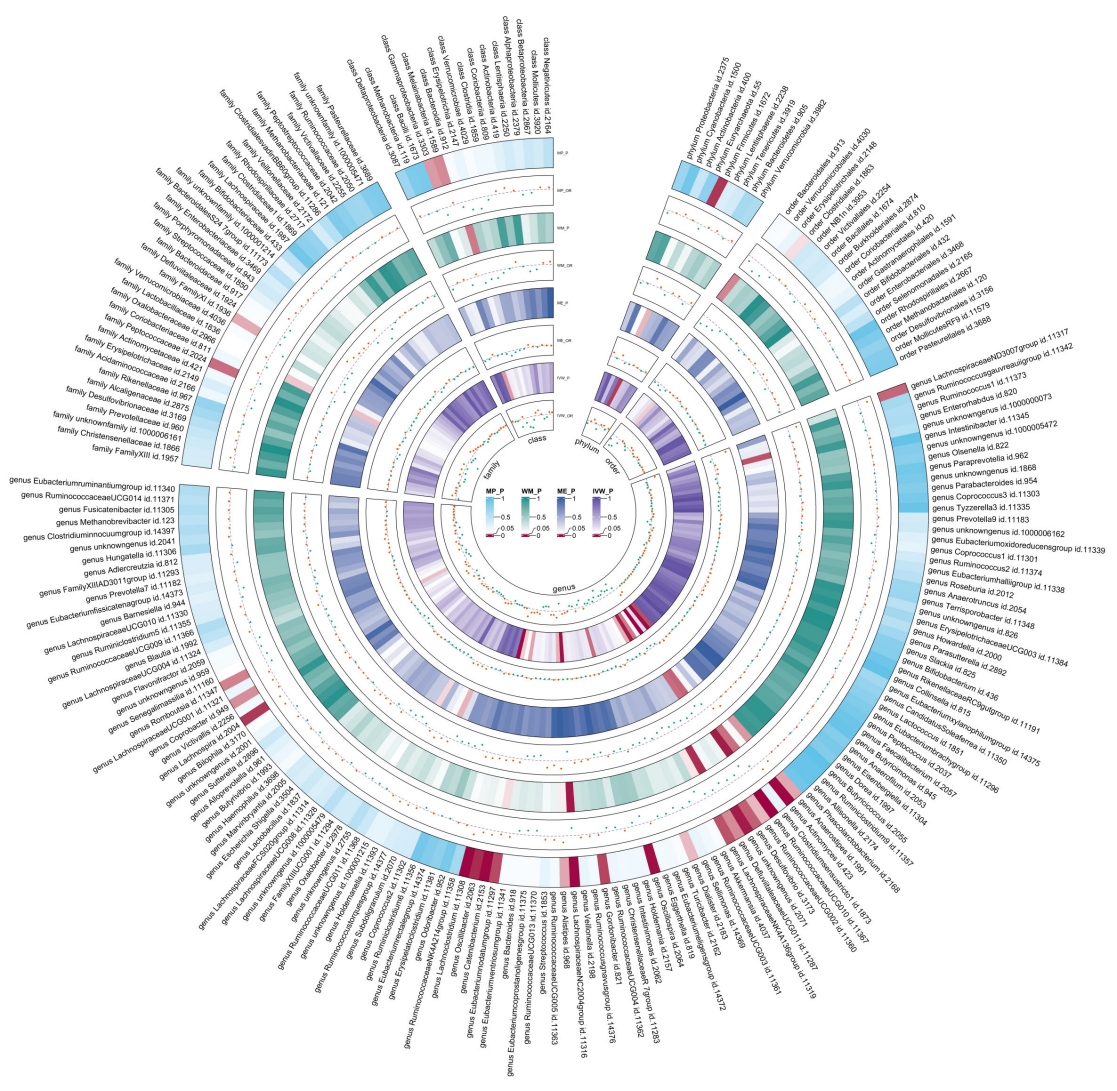
Circular heat map of meta-analysis of forward MR analysis between gut microbiota and T2DM. IVW, Inverse-Variance Weighted; ME, MR-Egger; WM, Weighted median; MP, MR-PRESSO. The color variations represented the size of the p-value. The scatter plots reflect OR, with OR > 1 labeled red and OR < 1 labeled green.

After the IVs selection procedure, one bacterial taxon (order *Lactobacillales*) was excluded from the meta-analysis due to significant pleiotropy. Then, meta-analyses of 210 gut bacteria were conducted, including 15 unknown taxa (**Supplementary Table 4**). Finally, we identified two bacterial taxa significantly associated with T2DM, and eight bacterial taxa suggestively associated with T2DM. The combined results of IVW method revealed that genetic predisposition to genus *RuminococcaceaeUCG010* (OR = 0.857, 95% CI 0.795, 0.924; P = 6.33×10^−5^, Rg_P = 1.19×10^−3^) and order *Clostridiales* (OR = 0.936, 95% CI 0.878, 0.997; P = 0.039, Rg_P = 0.010) were significantly and other four bacterial taxa were suggestively associated with a decreased risk of T2DM. Additionally, we found that genetically predicted genera *Actinomyces* (OR = 1.113, 95% CI 1.046, 1.185; P = 7.89×10^−4^), *Alistipes* (OR = 1.095, 95% CI 1.007, 1.191; P = 0.033), *Anaerostipes* (OR = 1.091, 95% CI 1.020, 1.168; P = 0.011), and *Eubacteriumnodatumgroup* (OR = 1.036, 95% CI 1.003, 1.070; P = 0.030) were suggestively associated with an increased risk of T2DM (**Figure 3**). All sensitivity analyses confirmed the consistency of the reported associations. The Cochran Q test, used to assess SNP heterogeneity, found no significant heterogeneity in most MR estimates within the meta-analysis. Pleiotropy did not need to be considered in this study due to the removal of the MR estimates with significant pleiotropy. Most meta-analysis results showed no or only mild heterogeneity. All the combined estimates are depicted in **Figure 4**.

### 3.3 Reverse MR analysis between gut microbiota and T2DM

**Figure 5:**
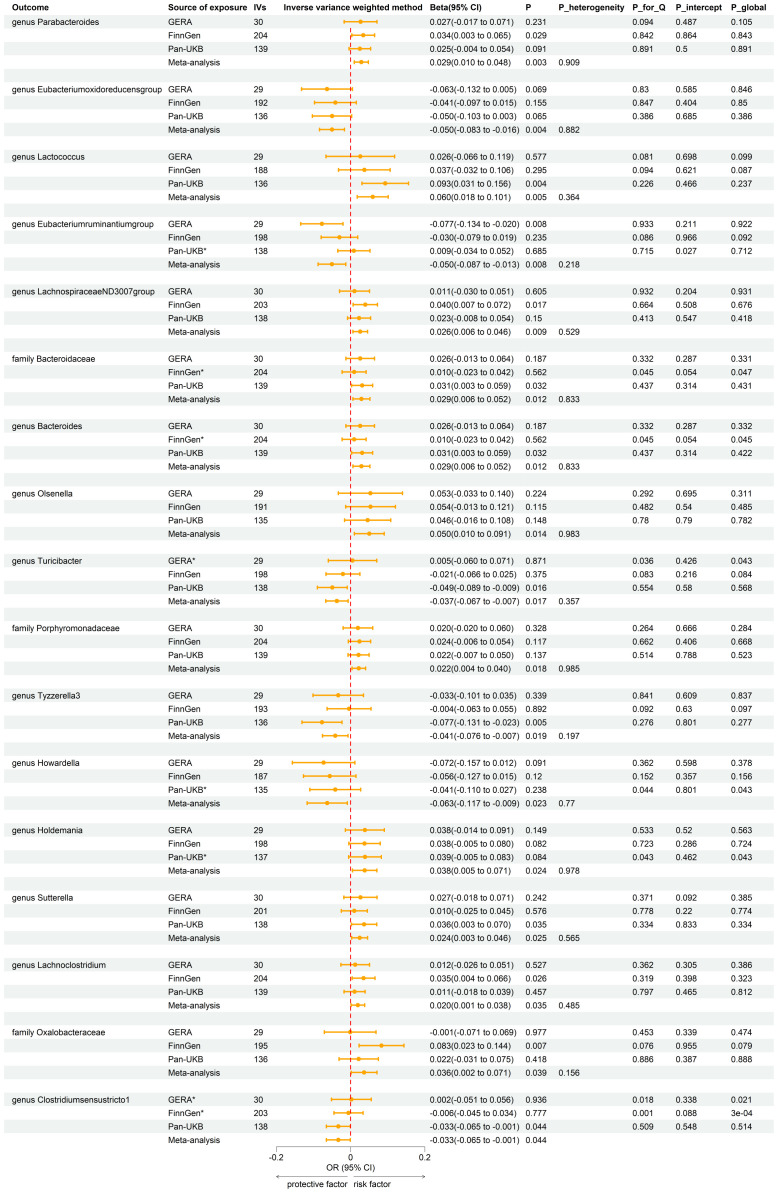
Forest plot of reverse MR analysis between gut microbiota and T2DM. IVs, instrumental variables; CI, confident interval; P_heterogeneity, p-value of heterogeneity for meta-analysis; P_for_Q, p-value for Cochran Q test; P_intercept, p-value for MR-Egger intercept test; P_global, p-value for Global test; *, excluded from the meta-analysis due to SNPs less than 4 or significant pleiotropy.

**Figure 6:**
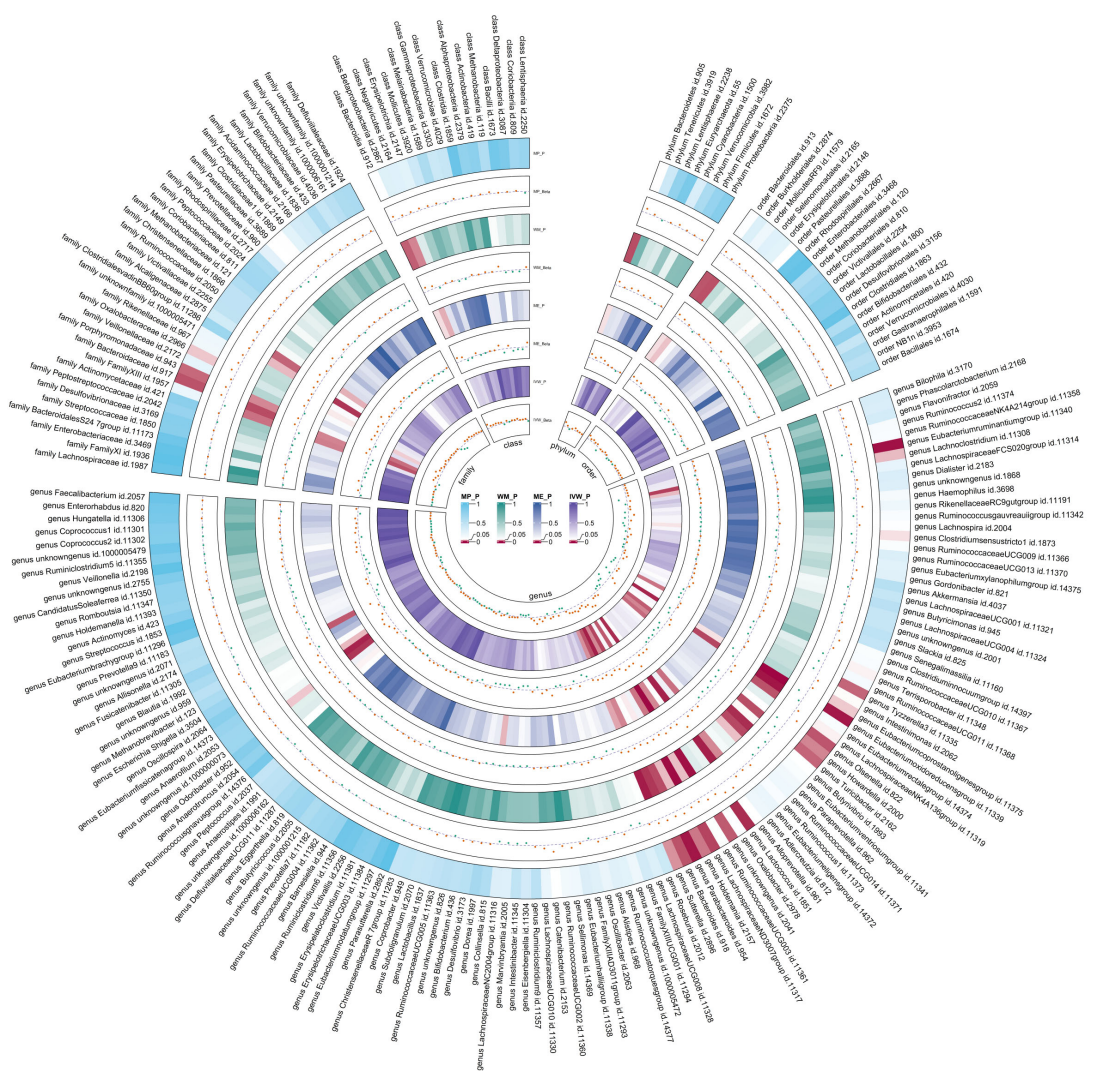
Circular heat map of meta-analysis of reverse MR analysis between gut microbiota and T2DM. IVW, Inverse-Variance Weighted; ME, MR-Egger; WM, Weighted median; MP, MR-PRESSO. The color variations represented the size of the p-value. The scatter plots reflect Beta, with Beta > 0 labeled red and Beta < 0 labeled green.

Using the same IVs selection procedure for gut microbiota, two taxa (genus *Coprococcus3* and phylum *Actinobacteria*) were excluded from the meta-analysis due to the significant IV pleiotropy of T2DM. Subsequently, 209 meta-analyses were performed, revealing a significant association between T2DM and four bacterial taxa, with a suggestive association for an additional 13 taxa (**Supplementary Table 5**). The combined results of IVW method revealed that genetically predicted T2DM was significantly associated with an increased risk of genus *Parabacteroides* (Beta = 0.029, 95% CI 0.010, 0.048; P = 0.003, Rg_P = 0.012), family *Porphyromonadaceae* (Beta = 0.022, 95% CI 0.004, 0.040; P = 0.018, Rg_P = 0.022), genus *Sutterella* (Beta = 0.024, 95% CI 0.003, 0.046; P = 0.025, Rg_P = 0.008), and genus *Lachnoclostridium* (Beta = 0.020, 95% CI 0.001, 0.038; P = 0.035, Rg_P = 0.005) (**Figure 5**). The abovementioned associations were consistent with all sensitivity analyses. The Cochran Q test revealed no heterogeneity in the MR estimates included in the meta-analysis. Our study design obviated the need to consider pleiotropy. Most meta-analysis results exhibited no or only mild heterogeneity. **Figure 6** displays all combined estimates. Bilateral MR analysis showed no bidirectional causality between gut microbiota and T2DM.

### 3.4 Forward MR analysis between plasma metabolites and T2DM, and MR analysis between identified gut microbiota and identified plasma metabolites

**Figure 7:**
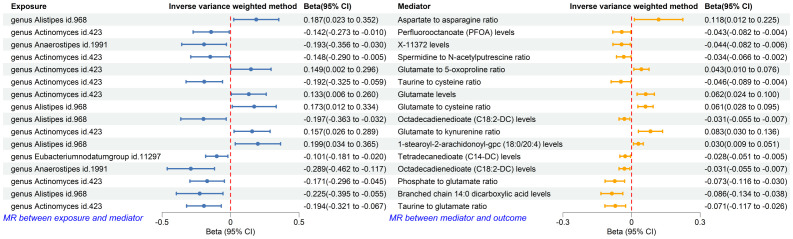
Forest plot of MR analysis between identified gut microbiota and identified plasma metabolites, and between identified plasma metabolites and T2DM. CI, confident interval.

The forward MR analysis identified 109 plasma metabolites genetically predicted to be suggestively causally associated with T2DM (**Supplementary Table 6**). Subsequently, 436 MR analyses were conducted between the four previously identified bacterial taxa and the 109 plasma metabolites (**Supplementary Table 7**). Among these, a genetic predisposition to the genus *Anaerostipes* was causally linked to lower levels of octadecadienedioate (C18:2-DC) (Beta = −0.289, 95% CI −0.462, −0.117; P = 0.001) and another plasma metabolite. The genus *Actinomyces*, as genetically predicted, was causally associated with the phosphate to glutamate ratio (Beta = −0.171, 95% CI −0.296, −0.045; P = 0.008), the taurine to glutamate ratio (Beta = −0.194, 95% CI −0.321, −0.067; P = 0.003), and six other plasma metabolites. Genetically predicted genus *Alistipes* was causally associated with branched chain 14:0 dicarboxylic acid levels (Beta = −0.225, 95% CI −0.395, −0.055; P = 0.010) and four other plasma metabolites. The genetically predicted genus *Eubacteriumnodatumgroup* was causally linked to a decreased risk of a specific plasma metabolite (**Figure 7**).

### 3.5 Mediation analysis

Using the product of coefficients method, we calculated the IE of 16 identified pairs of gut microbiota and plasma metabolites (**Supplementary Table 8**). Specifically, the genus *Anaerostipes* indirectly influenced T2DM through octadecadienedioate levels, with an IE of 0.009 (95% CI 0.0003, 0.0177; P = 0.043) and a mediated proportion of 10.29%. The genus *Actinomyces* indirectly impacted T2DM through the phosphate to glutamate ratio, with an IE of 0.0125 (95% CI 0.0007, 0.0242; P = 0.039), and the taurine to glutamate ratio, with an IE of 0.0138 (95% CI 0.0012, 0.0264; P = 0.037); the mediated proportions were 11.68% and 12.90%, respectively. The genus *Alistipes* indirectly influenced T2DM through branched chain 14:0 dicarboxylic acid levels, with an IE of 0.0138 (95% CI 0.0012, 0.0264; P = 0.032) and a mediated proportion of 21.19% (**Table 3**).

**Table 3:** Mediation analysis between gut microbiota, plasma metabolites, and T2DM.

Gut microbiota	Metabolite	Total effect EO (95% CI)	Effect EM (95% CI)	Effect MO (95% CI)	Indirect effect (95% CI)	Mediated proportion
genus Anaerostipes	Octadecadienedioate (C18:2-DC) levels	0.0875 (0.02, 0.155)	-0.2894 (-0.4619, -0.1169)	-0.031 (-0.0547, -0.0074)	0.009 (0.0003, 0.0177) P=0.043	10.29%
genus Actinomyces	Phosphate to glutamate ratio	0.107 (0.0445, 0.1694)	-0.1706 (-0.2962, -0.045)	-0.0731 (-0.1158, -0.0304)	0.0125 (0.0007, 0.0242) P=0.039	11.68%
genus Alistipes	Branched chain 14:0 dicarboxylic acid levels	0.0911 (0.0073, 0.1749)	-0.2252 (-0.3955, -0.0548)	-0.0859 (-0.1338, -0.038)	0.0193 (0.0012, 0.0375) P=0.037	21.19%
genus Actinomyces	Taurine to glutamate ratio	0.107 (0.0445, 0.1694)	-0.1939 (-0.3205, -0.0673)	-0.0712 (-0.1167, -0.0257)	0.0138 (0.0012, 0.0264) P=0.032	12.90%

EO, exposure to outcome; EM, exposure to mediator; MO, mediator to outcome.

## 4 Discussion

In our study, significant negative genetic correlations were identified between T2DM and specific gut microbiota, particularly the genus *RuminococcaceaeUCG010* and order *Clostridiales*. These taxa were also causally associated with a decreased risk of T2DM, suggesting a protective effect against the disease. Additionally, the study highlighted plasma metabolites as mediators in the relationship between gut microbiota and T2DM. Specific metabolites, such as octadecadienedioate and branched chain 14:0 dicarboxylic acid, were implicated in these interactions. For instance, the genus *Anaerostipes* was associated with a decreased risk of octadecadienedioate levels, indirectly affecting T2DM risk. These findings provide valuable insights into the potential pathways through which gut microbiota influence T2DM and highlight the role of plasma metabolites as critical mediators.

Our research findings align with emerging theories on the impact of specific gut microbiota on metabolic diseases, particularly T2DM. An MR analysis identified a causal relationship between *RuminococcaceaeUCG010* and a reduced risk of T2DM. These bacteria produce short-chain fatty acids (SCFAs) like butyrate and propionate, which enhance gut barrier function, modulate inflammation, and improve insulin sensitivity (9). Another study found that genera like *RuminococcaceaeUCG010* significantly influence glycemic responses to treatments in T2DM patients, highlighting their role in metabolic health and diabetes management (30). Additionally, species like *Clostridium butyricum*, known for butyrate production, have shown in diabetic mouse models to improve diabetes markers, supporting their beneficial role in managing hyperglycemia and metabolic dysfunctions (31).

Previous studies have identified that *Actinomyces* species, such as *Streptomyces*, can produce significant amounts of glutamic acid, a key neurotransmitter and metabolic intermediate in both bacterial and human cells (32). This aligns with our findings, which showed that the genus *Actinomyces* might decrease the phosphate to glutamate ratio and the taurine to glutamate ratio by elevating glutamic acid levels. *Anaerostipes* are known for producing SCFAs through the fermentation of dietary fibers, potentially influencing various metabolic intermediates, including octadecadienedioate (33, 34). Similarly, the genus *Alistipes* has been implicated in the metabolism of branched-chain fatty acids (BCFAs). *Alistipes* species possess unique enzymatic capabilities that allow them to interact with complex lipid molecules, potentially leading to the observed decrease in branched-chain 14:0 dicarboxylic acid levels (35). However, the specific mechanisms by which *Anaerostipes* decrease octadecadienedioate levels and *Alistipes* decrease branched-chain 14:0 dicarboxylic acid levels were not detailed in the available literature. Although we demonstrated a positive causal relationship, further exploration is necessary to clarify these mechanisms.

Our study showed that decreased phosphate to glutamate and taurine to glutamate ratios could increase T2DM risk, likely due to elevated glutamate levels. Glutamate acts as an intracellular messenger in pancreatic β-cells, linking glucose metabolism to insulin exocytosis. However, excessive intracellular glutamate can inhibit insulin secretion by disrupting calcium signaling necessary for insulin granule release. Lehtihet et al. found that increased L-glutamate levels inhibit protein phosphatases, promoting insulin exocytosis in a Ca^2+^-independent manner. This dysregulation can initially cause excessive insulin secretion, followed by β-cell exhaustion and decreased insulin output (36). Excessive glutamate can also cause excitotoxicity, leading to β-cell death or dysfunction by overactivating glutamate receptors and subsequent calcium influx, triggering apoptotic pathways. Maechler et al. highlighted that while intracellular glutamate amplifies insulin secretion, extracellular glutamate can activate ionotropic receptors, slowing insulin exocytosis and contributing to β-cell dysfunction (37). Octadecadienedioate is a metabolite linked to fatty acid metabolism. Changes in its levels can reflect disturbances in fatty acid metabolism, common in T2DM patients (38). Decreased octadecadienedioate levels can disrupt fatty acid metabolism, leading to lipid accumulation, inflammation, mitochondrial dysfunction, altered lipid signaling, and genetic/epigenetic changes, collectively impairing insulin sensitivity and increasing T2DM risk. For example, decreased octadecadienedioate levels can lead to lipid accumulation in non-adipose tissues like liver and muscle, where excess lipids activate serine/threonine kinases like PKC, which phosphorylate insulin receptor substrate-1 (IRS-1) on serine residues, impairing IRS-1’s ability to activate downstream insulin signaling, reducing glucose uptake by cells (39). Additionally, decreased octadecadienedioate levels can disrupt mitochondrial function, impairing mitochondrial fatty acid oxidation and resulting in diacylglycerol (DAG) and ceramide accumulation, which activate PKC and other kinases that impair insulin signaling (40). Branched-chain 14:0 dicarboxylic acid, a BCFA with 14 carbon atoms and two carboxyl groups, is of interest due to its unique metabolic pathways and potential health implications. BCFAs are implicated in enhancing insulin sensitivity. Studies show that higher levels of BCFAs, like odd-chain fatty acids (C15:0 and C17:0), are associated with better insulin sensitivity and lower T2DM risk. These fatty acids improve insulin signaling pathways, facilitating better glucose uptake by cells and reducing blood glucose levels (41). Additionally, BCFAs regulate glucose and lipid metabolism, enhancing mitochondrial function and fatty acid oxidation, maintaining energy balance, and preventing lipid accumulation in tissues, a common issue in T2DM. Enhanced lipid metabolism through BCFAs reduces lipotoxicity and improves insulin action (42). This aligns with our observations that reduced levels of branched-chain 14:0 dicarboxylic acid are associated with increased T2DM risk. Understanding how these metabolites interact with metabolic and cellular processes can help elucidate T2DM pathophysiology and identify new therapeutic targets.

To elaborate on the impact of metabolic disturbances on overall health, maintaining gut eubiosis is crucial for managing T2DM and enhancing overall well-being. A diet rich in fiber, prebiotics, and probiotics supports the growth of beneficial gut bacteria. Foods such as fruits, vegetables, whole grains, and fermented products like yogurt and kefir are particularly beneficial (43). Reducing the intake of processed foods, sugars, and artificial additives helps maintain a balanced gut microbiota (44). Regular physical activity, adequate hydration, and stress management are important lifestyle factors that contribute to gut health (45). Taking probiotic supplements can provide additional support, especially after antibiotic treatments that disrupt gut microbiota balance (46). These interventions promote a healthy and diverse gut microbiota, essential for overall health and well-being.

This study robustly applies MR and LDSC to elucidate the complex interplay between gut microbiota, plasma metabolites, and T2DM. A primary strength of this approach is the significant reduction in confounding factors, enhancing the reliability of causal inferences. The comprehensive use of multiple outcome sources and sensitivity analyses further substantiates the robustness of our findings, mitigating potential biases such as pleiotropy and population stratification. However, the study’s strength lies not only in its current findings but also in setting a foundation for future research. Future studies should include a broader range of ethnic groups, as our data primarily comes from individuals of European descent. Expanding to other populations would help determine if these genetic associations hold universally. While our study identifies significant associations and potential pathways, it does not fully unravel the biological mechanisms at play. Employing cutting-edge technologies like single-cell RNA sequencing and advanced computational models could uncover the microbial species and their roles in T2DM.

Assessing the results of our study requires an understanding of its limitations. Firstly, the GWAS data related to gut microbiota were collected from 18,340 participants across various ethnic backgrounds. However, the T2DM GWAS summary statistics were derived exclusively from individuals of European descent. This discrepancy could limit the generalizability of our findings across different ethnicities and demographic groups. Though nearly 80% of the gut microbiota data from these GWAS were from European populations, further research involving diverse populations is necessary to validate our results and ensure broader applicability. Secondly, although MR methods help reduce confounding and reverse causation, potential biases still exist. For instance, pleiotropy, where genetic variants influence multiple traits, could bias the causal estimates. We used MR-Egger and MR-PRESSO to detect and adjust for pleiotropy, but these methods have limitations and may not entirely eliminate pleiotropic effects. Thirdly, there are slight discrepancies between different datasets. However, the overall heterogeneity remains minimal, confirming the stability and reliability of our results. Lastly, the lack of detailed data precluded stratified analyses by age and gender, inhibiting our ability to explore potential differences across various demographics.

Our findings contribute to the growing research on the gut microbiota’s role in metabolic diseases, particularly T2DM. They support theories suggesting that specific gut microbiota influence metabolic health through various mechanisms. For instance, the protective role of *RuminococcaceaeUCG010* and *Clostridiales* in reducing T2DM risk supports studies indicating their involvement in enhancing gut barrier function and improving insulin sensitivity. Our study also highlights new metabolic pathways, such as those involving octadecadienedioate and branched-chain 14:0 dicarboxylic acid, through which gut microbiota may influence T2DM. This expands our understanding of the interplay between microbial metabolites and host metabolism, suggesting potential targets for therapeutic interventions. Modulating specific gut bacteria to alter metabolite levels could improve metabolic outcomes. These findings both align with and challenge current theories. They corroborate the role of gut microbiota in metabolic regulation while emphasizing the importance of considering specific microbial taxa and their metabolic products in T2DM pathogenesis. This nuanced perspective can guide future research and clinical strategies aimed at preventing and managing T2DM through microbiota-based interventions.

## 5 Conclusion

Our study highlights significant genetic correlations between gut microbiota and T2DM, mediated through plasma metabolites. The identification of specific microbial taxa, such as genus *RuminococcaceaeUCG010* and order *Clostridiales*, as protective factors against T2DM, underscores their potential as therapeutic targets. This research advances the field by elucidating the metabolic pathways linking gut microbiota to T2DM, paving the way for microbiota-based interventions. Future research should validate these findings across diverse populations and employ advanced techniques like single-cell RNA sequencing to further explore the biological mechanisms involved. Our results advocate for the development of therapeutic strategies targeting gut microbiota to improve metabolic health and manage T2DM, offering new avenues for personalized medicine and dietary interventions.

## Data Availability

The original contributions presented in the study are included in the article/**Supplementary Material**. Further inquiries can be directed to the corresponding author.
